# Identification of nephropathy candidate genes by comparing sclerosis-prone and sclerosis-resistant mouse strain kidney transcriptomes

**DOI:** 10.1186/1471-2369-13-61

**Published:** 2012-07-19

**Authors:** Ashraf El-Meanawy, Jeffery R Schelling, Sudha K Iyengar, Patrick Hayden, Shrinath Barathan, Katrina Goddard, Fatima Pozuelo, Essam Elashi, Viji Nair, Matthias Kretzler, John R Sedor

**Affiliations:** 1 Department of Medicine, MetroHealth System, Case Western Reserve University, Cleveland, OH, USA; 2 Department of Epidemiology and Biostatistics, Case Western Reserve University, Cleveland, OH, USA; 3 Department of Medicine, University of Michigan, Ann Arbor, MI, USA; 4 Kidney Disease Center, Medical College of Wisconsin, Milwaukee, WI, USA

## Abstract

**Background:**

The genetic architecture responsible for chronic kidney disease (CKD) remains incompletely described. The Oligosyndactyly (*Os*) mouse models focal and segmental glomerulosclerosis (FSGS), which is associated with reduced nephron number caused by the *Os* mutation. The *Os* mutation leads to FSGS in multiple strains including the ROP-*Os*/+. However, on the C57Bl/6J background the mutation does not cause FSGS, although nephron number in these mice are equivalent to those in ROP-*Os*/+ mice. We exploited this phenotypic variation to identify genes that potentially contribute to glomerulosclerosis.

**Methods:**

To identify such novel genes, which regulate susceptibility or resistance to renal disease progression, we generated and compared the renal transcriptomes using serial analysis of gene expression (SAGE) from the sclerosis-prone ROP-*Os*/+ and sclerosis resistant C57-*Os*/+ mouse kidneys. We confirmed the validity of the differential gene expression using multiple approaches. We also used an Ingenuity Pathway Analysis engine to assemble differentially regulated molecular networks. Cell culture techniques were employed to confirm functional relevance of selected genes.

**Results:**

A comparative analysis of the kidney transcriptomes revealed multiple genes, with expression levels that were statistically different. These novel, candidate, renal disease susceptibility/resistance genes included neuropilin2 (*Nrp2*), glutathione-S-transferase theta (*Gstt*1) and itchy (*Itch*). Of 34 genes with the most robust statistical difference in expression levels between ROP-*Os/*+ and C57-*Os/+* mice, 13 and 3 transcripts localized to glomerular and tubulointerstitial compartments, respectively, from micro-dissected human FSGS biopsies. Network analysis of all significantly differentially expressed genes identified 13 connectivity networks. The most highly scored network highlighted the roles for oxidative stress and mitochondrial dysfunction pathways. Functional analyses of these networks provided evidence for activation of transforming growth factor beta (TGFβ) signaling in ROP-*Os/*+ kidneys despite similar expression of the TGFβ ligand between the tested strains.

**Conclusions:**

These data demonstrate the complex dysregulation of normal cellular functions in this animal model of FSGS and suggest that therapies directed at multiple levels will be needed to effectively treat human kidney diseases.

## Background

Focal segmental glomerulosclerosis (FSGS) is a leading cause of nephrotic syndrome in adults and is particularly prevalent in the African American population [1-3]. The incidence of FSGS is increasing in young children [4,5]. Immunosuppressive therapies are the mainstay of treatment, though most patients with FSGS are resistant to treatment [6]. Unfortunately, a large proportion of FSGS patients, who do not respond to treatment, frequently continue to lose kidney function and progress to end stage renal disease (ESRD), requiring dialysis or kidney transplantation. Moreover, recurrent FSGS is a leading cause of proteinuria and allograft loss after kidney transplantation [7-9].

In recent years, genes have been linked or associated with both familial and sporadic FSGS [10-16], which supports the premise that genetic factors play a major pathophysiologic role [17]. To identify novel, candidate FSGS susceptibility genes, we used Serial Analysis of Gene Expression (SAGE) to characterize the kidney transcriptome in the Oligosyndactyly (*Os*) mouse, a genetic model of oligomeganephronia and progressive FSGS. Homozygous *Os/Os* mice die *in utero* at the 64-cells stage of development [18], but heterozygote *Os/+* mice have a skeletal phenotype mainly in the form of fused digits (oligosyndactyly), and a renal phenotype in the form of 50% reduction in nephron number [19,20]. The ROP/GnLe strain (ROP-*Os*/+), was established at the Jackson Lab and has been maintained by sibling mating. As the ROP-*Os*/+ mice age, they develop a histopathologic lesion similar to human FSGS. The *Os* mutation causes on average 50% reduction of nephron number, an effect which is independent of genetic background. The ROP-*Os/+* mice model progressive kidney disease, as demonstrated by glomerulosclerosis, proteinuria, and increase in creatinine which reflects a diminished glomerular filtration rate ( [21], and El-Meanawy, unpublished data). The *Os* mutation produces a similar renal phenotype when bred on other genetic backgrounds, such as FvB and C3H [20,22-24]. However, a congenic mouse strain, the C57Bl/6J-*Os*/+ (C57-*Os*/+), which has the *Os* mutation on a C57Bl/6J background, develops oligosyndactyly and oligomeganephronia but not FSGS [22], thus providing an ideal model for transcriptome comparison with the ROP-*Os/+* mouse.

SAGE captures an unbiased, quantitative snapshot of gene expression patterns [25-27]. SAGE libraries are comprised of cDNA sequence tags derived from the 3’ end of the cDNA pool, and a count of SAGE-derived cDNA tags provides a quantitative representation of the corresponding mRNAs in the sample. We previously used SAGE to generate a tag library from normal mouse kidney, which yielded a potassium channel not known to be expressed in the kidney [28,29]. In the current report, we exploited phenotypic differences between the sclerosis-prone ROP-*Os*/+ and the sclerosis-resistant C57-*Os*/+ mice to discover candidate FSGS susceptibility and/or protective genes by comparing the kidney transcriptomes between the two mouse strains using SAGE. To identify pathophysiologically relevant genes, we specifically examined transcriptome profiles in young mice after completion of nephrogenesis, but prior to the development of significant FSGS. Statistical analysis of the SAGE libraries from ROP-*Os*/+ and C57-*Os*/+ mice kidneys, followed by assembly of differentially expressed transcripts into networks, allowed identification of genes which potentially play a role in the pathogenesis of glomerulosclerosis in the *Os* mouse model.

## Methods

### Animals and tissue collection

Animal experiments were conducted in accordance with the National Institute of Health guidelines and approved by the Institutional Animal Care and Utilization committee at Case Western Reserve University and the Medical College of Wisconsin. ROP-*Os*/+ and C57-*Os*/+ mice were purchased from Jackson Laboratories (Bar Harbor, ME) and housed in the animal facilities at the above institutions. We phenotyped mice at 6,10,12, and 16 weeks by comparing urine protein to creatinine ratio,glomerular surface area, and sclerosis score in Periodic Acid Schiff (PAS) stained sections as previously described [30]. Glomerular number was determined by the maceration method as previously described [31]. The kidneys were collected for SAGE library construction at 6 weeks of age to avoid identification of developmental genes (because mouse nephrogenesis continues up to 4 weeks of age), and prior to the onset of significant renal disease. After sacrifice, the kidneys were promptly removed and the renal medulla was dissected away and discarded. A small portion of each kidney was fixed in formaldehyde, sectioned, and stained with PAS for histopathological analysis.

### Generation of SAGE libraries

Poly(A)^+^ RNA was isolated from renal cortices of ROP-*Os/+* and C57Bl/6-*Os/+* mice using ion-exchange column kits from Qiagen (Valencia, CA). The quality of the isolated RNA was analyzed by agarose gel electrophoresis and RNA concentration was determined by UV spectrophotometry [32]. RNA from three mice per strain was pooled and cDNA was synthesized from a starting amount of 5 μg poly(A)^+^ RNA using cDNA synthesis kit, according to manufacturer recommendation (Invitrogen). A fraction of first and second strand reactions was labeled with ^32^P] to evaluate synthesis efficiency. cDNA quality was assessed using agarose gel electrophoresis and by measuring radioisotope incorporation. SAGE steps were performed as described previously [29] using the restriction endonuclease *Nla*-III as the anchoring enzyme [29]. We used SAGE software to analyze the SAGE produced concatemer sequences, which identifies the cDNA tags flanked by* Nla*-III restriction enzyme recognition sequence. In addition, the software identifies and excludes duplicate ditags, which are likely to be PCR amplification artifacts. Confirmation of SAGE tag gene identity, as well as relative expression levels were performed using northern blots, RNase protection assay or real-time RT-PCR. The confirmatory methods and data are in a Additional file 1.

### Statistical analysis of SAGE tags

We applied several statistical models, which employ Bayesian methods or Poisson distributions, to identify tags that were significantly different between the ROP-*Os*/+ and C57-*Os*/+ kidney libraries [33-36]. The method allows comparison of unequal libraries based on the assumption that tag counts are binomially distributed. Regardless of the method used to identify statistically significant differentially expressed tags, rank orders and p-values were comparable.

### Annotation of SAGE tags

Accurate annotation of SAGE tag libraries requires a combination of methods. Accordingly, we used multiple approaches to annotate SAGE tags including the online engine “SAGE genie” ( http://cgap.nci.nih.gov/SAGE/mSEM), and manual nucleotide blast (blastn) searches. We also constructed genome-wide mouse cDNA data sets from NCBI-Ref-Seq and Riken-Fantom collections and designed a software program, which identifies and catalogues the 3’-most “CATG” sequence string (*Nla*-III recognition sequence) with the 3’ flanking 10 nucleotides. These tag-to-gene database tables were used to annotate the SAGE tags. Ambiguous tags, which mapped to multiple genes, and single copy tags were excluded. Tags were assigned gene identifiers if the tag mapped to a sequence that contained the most 3’-CATG sequence. Sequence databases were searched in the following priority: 1) our own libraries containing tags extracted from Ref-Seq and Fantom cDNA databases; 2) the mouse mSAGE expression matrix using SAGE Genie; or 3) the mouse mRNA (non-redundant [nr] and expressed sequence tags [dbest]) databases using blastn.

### Assembly of transcriptome networks and pathways

We utilized the Ingenuity Pathway Analysis (IPA) system (Ingenuity Systems, Mountain View, CA, USA, http://www.ingenuity.com) to visualize the SAGE data as biological networks. To perform a “connectivity” analysis, a table containing selected gene identifiers, defined as Focus Genes, and their corresponding relative mRNA expression ratios between ROP-*Os/+* and C57-*Os/+* kidneys was uploaded into the application. Criteria for selection as Focus Genes included: 1) a greater than 2-fold difference in expression levels between the ROP-*Os/+ *and C57-*Os/+* kidney libraries; and/or 2) a p-value < 0.05 for differential expression after correction for multiple testing. Three hundred and nine genes met these criteria, were mapped to their corresponding gene objects in the Ingenuity Pathway Knowledge Base and subsequently overlaid onto a global molecular network developed from information contained in Ingenuity Pathways Knowledge Base. Networks were then algorithmically generated based on the connectivity of the eligible genes. Twenty-eight Focus Genes failed to generate a shared molecular network or pathway. Function of the connectivity networks was inferred from the functions of the genes in the network as annotated in the Knowledge Base.

An additional set of networks was generated using the functional information in the Ingenuity Pathways Knowledge Base (“functional” analysis). Genes from the SAGE kidney libraries, which met the cutoffs defined above and that were associated with biological functions in the Ingenuity Pathways Knowledge Base, were considered for this analysis. Right-tailed Fisher’s exact test was used to calculate a p-value reflecting the probability that each biological function assigned to that network is due to chance alone. Networks were ranked according to the combinatorial p-value of differentially expressed genes represented in the network.

### TGFβ Western blot analysis

Western blot of kidney protein extracts were performed as previously described [37] using *TGFβ* specific antibody. Equal loading was verified by stripping the membrane and probing for actin expression.

### TGFβ assay

TGFβ activity was determined using mink lung epithelial cells expressing the firefly luciferase reporter gene under the control of the minimal, TGFβ-responsive, plasminogen activator inhibitor-1 (PAI-1) promoter [38] (gift from Dr. Daniel Rifkin, New York University). The culture conditions were as previously described [39]. The effect of *Itch* overexpression on TGFβ was determined from two nearly confluent six-well tissue culture plates, which were transiently transfected with either mouse *Itch* cDNA which was cloned in pCDNA3.1 or empty vector control, using lipofectamine (Invitrogen) according to manufacturer recommendations. Twenty-four hours after transfection, TGFβ (R&D system) was added to the culture media at 200 ng/ml. The cultures were maintained for 8 h and luciferase activity was assayed using a Promega kit according to manufacturer instructions. Relative luminescence units were determined compared to control conditions (empty pCDNA3.1 plasmid transfected cells).

### Comparing differentially expressed Os mice genes to human FSGS expression data

We compared 40 SAGE-derived transcripts with the most divergent expression levels between ROP-*Os/+* versus C57-*Os/+* kidneys, with the human microarray expression data, comprised of samples obtained from micro-dissected human kidney biopsies from patients with FSGS [40]. The fold change and significance (assessed by q-value, corrected for multiple testing) was calculated by the Significance Analysis of Microarrays method (SAM) [41] using the MeV software from the Institute of Genome Research ( http://www.jcvi.org).

### Identification of glomerular enriched genes in the Os model of FSGS

To identify genes which are differentially expressed in our model system, and are known to be expressed in the glomerulus, we searched for common genes between the glombase [42] gene list, SAGE glomerular tags [43] and annotated transcripts with the most significant differential expression between ROP-*Os/+* versus C57-*Os/+* kidneys. We then used Pubmed and Ingenuity pathway analysis to mine the literature for possible links between these genes and nephropathy.

## Results

### Mouse phenotype

We found that the urine protein to creatinine ratio was significantly higher in ROP-*Os/+* mice compared to both C57-*Os/+* and ROP-+/+ at all time points. The proteinuria in ROP-*Os/+* mice reached a peak at 12 weeks. The glomerular surface area at 6 weeks was similar between the ROP-*Os/+* and C57-*Os/+* (5646 ± 286 and 5708 ± 460 μm^2^, respectively) and significantly larger than glomerular surface area of ROP-+/+ (3984 ± 456 μm^2^). However, by 16 weeks of age, the surface area had increased significantly in the ROP-*Os/+* compared to C57-*Os/+*glomeruli (8990 ± 628 vs 6458 ± 1259 μm^2^). Furthermore, the ROP-*Os/+ *mice showed progressive increase in sclerosis score over time (Additional file 1).

### Os/+ kidney SAGE libraries

We constructed SAGE libraries from renal cortical RNA extracted from ROP-*Os*/+ and C57-*Os*/+ kidneys at 6 weeks of age (Table 1), a time point which permitted completion of differentiation, but prior to observation of significant glomerulosclerosis. The combined ROP-*Os*/+ and C57-*Os*/+ kidney SAGE libraries contained 49,588 cDNA sequence tags, of which 20,594 were unique. We did not observe “loss of AT-rich tags” bias [44] and the libraries demonstrated 54.32% A+T and 45.68% C+G nucleotides. Table 2 shows the 50 most statistically significant, differentially expressed and annotated tags. We compared the annotated genes in our SAGE libraries to publically available wild type mouse *Nla*-III-anchored SAGE libraries. Of the genes not previously described in SAGE kidney libraries, we observed significant expression of arginine-glutamic acid dipeptide (RE) repeats (*Rere* or *Atrophin*-2), insulin-like growth factor binding protein (*Igfbp*7), aminolevulinic acid synthase (*Alas*1), glutathione S-transferase, theta 1 (*Gstt*1), and propionyl coenzyme A carboxylase (*Pccb*). Interestingly, all of these transcripts were induced to a detectable level in ROP-*Os/+*, suggesting that deep SAGE coverage can unmask changes in kidney gene expression patterns induced in a disease model. To identify differentially expressed glomerular genes, we searched for common genes between glombase [42], SAGE glomerular tags [43] and 220 annotated transcripts with the most significant differential expression between ROP-*Os/+* versus C57-*Os/+* kidneys. This comparison yielded 67 genes, of which 53 were upregulated, and only 14 were downregulated in the ROP-*Os/+* mouse (Table 3). We confirmed a reduction in glomerular number in 6 week old ROP-*Os*/+ mice (data not shown), suggesting that upregulated gene expression is not due to altered glomerular mass.

**Table 1:** SAGE tag counts

	**C57-Os/+**	**ROP-Os/+**
Total tags	26599	22989
Unique tags	11014	9580

The statistical analysis algorithm normalizes the tag representation in each library, based on the total number of tags per library. Duplicate ditags and tags that contained linker sequence were excluded from the analysis.

**Table 2:** **Top 50 differentially expressed tags between ROP-*Os/+ *and C57-*Os/+*kidneys**

**TAG**	**ROP-Os**	**C57-Os**	**P-Value**	**Gene Symbol**	**Gene Name**
CTATCCTCTC	873	613	1.47E-10	Gpx3	Glutathione peroxidase 3
ATTAACTTGG	54	14	75E-06	Glud1	Glutamate dehydrogenase 1
TGGTTGCTGG	8	42	2.53E-05	Nrp2b	Neuropilin2b
TCAAAAAAAA	15	0	0.000115878	Pea15	Phosphoprotein enriched in astrocytes 15
ACAAAAAAAA	20	2	0.000138392	Pde6c	Phosphodiesterase 6C, cGMP specific, cone, alpha prime
AACTTGATTA	14	0	0.000237608	Ndufa12	NADH dehydrogenase (ubiquinone) 1 alpha subcomplex, 12
GTTCACTTTC	27	6	0.000390333	Atp5e	ATP synthase, H+ transporting, mitochondrial F1 complex, epsilon subunit
CTGCTGTAAT	15	2	0.000577048	Aspm	Asp (abnormal spindle)-like, microcephaly associated (Drosophila)
TGTTGTGTTT	0	15	0.000858214	Lman2l	Lectin, mannose-binding 2-like
GTGAGCCCAT	0	14	0.001280824	Hsp90ab1	Heat shock protein 90 kDa alpha (cytosolic), class B member 1
GCCACGCCCC	24	6	0.001296626	Hpd	4-hydroxyphenylpyruvic acid dioxygenase
ATTCTCCAGT	14	39	0.0017137	Rpl23	Ribosomal protein L32
GTCCTGAGAG	9	0	0.002106134	Vamp8	Vesicle-associated membrane protein 8
ATAAAAAAAA	9	0	0.002106134	Bag4	BCL2-associated athanogene 4
ATTCTAACAT	15	2	0.002151531	Acadm	Acyl-Coenzyme A dehydrogenase, medium chain
TGGATGCCTT	1	16	0.002228166	Adh1	Alcohol dehydrogenase
GATTCCGTGA	1	4	0.002494576	Rpl37	Ribosomal protein L37
GCTTTGAATG	19	4	0.002494576	Atpif1	ATPase inhibitory factor 1
TGTCATCTAG	6	30	0.000282	Rpsa	Ribosomal protein SA (laminin receptor like 1)
TGCTGCTCCC	0	12	0.00287027	Gyk	Glycerol kinase
GCTGGCCTCC	1	15	0.003314916	Rhoq	Ras homolog gene family, member Q
GCCAAGTGGA	22	6	0.0041826	Eef2	Eukaryotic translation elongation factor 2
GTTTGTAAAA	22	6	0.0041826	Acsm3	Acyl-CoA synthetase medium-chain family member 3
AGATAACACA	8	0	0.004417209	Rere (atrophin-2)	Arginine glutamic acid dipeptide (RE) repeats
AAGACCTATG	39	17	0.004902036	Dbi	Diazepam binding inhibitor
ATCCGATTCC	11	31	0.005368014	Miox	myo-inositol oxygenase
GTCAATGACG	1	13	0.007371123	Aqp1	Aquaporin1
TCAGGCTGCC	180	130	0.008291421	Fth1	Ferritin heavy chain1
TTGTTAGTGC	36	66	0.008331722	Mdh1	Malate dehydrogenase 1
CTAGTCTTTG	22	7	0.008750438	Rps29	Ribosomal protein S29
CTGCTGTGGA	22	7	0.008750438	Hmgcs2	3-hydroxy-3-methylglutaryl-Coenzyme A synthase 2
AGAGACAAGG	46	23	0.008962126	Ndrg1	N-myc downstream regulated gene 1
GATCAGAAAA	7	0	0.009358827	Prdm16	PR domain containing 16
GTGTGATACA	7	0	0.009358827	Pccb	propionyl Coenzyme A carboxylase, beta polypeptide
CAGTTGGTTC	7	0	0.009358827	Mm.399814	Transcribed locus
CAGTAAAAAA	7	0	0.009358827	Map3k7ip1	Mitogen-activated protein kinase kinasekinase 7interacting protein 1
AACTTTTAAA	7	0	0.009358827	Hp1bp3	Heterochromatin protein 1, binding protein 3
GCTGTATTCA	7	0	0.009358827	Folh1	Folate hydrolase
AATAAAAACT	7	0	0.009358827	FBXL12	F-box and leucine-rich repeat protein 12
TTTGTGACTG	7	0	0.009358827	Ctbp1	C-terminal binding protein 1
AGATCTGCCC	7	0	0.009358827	Atp6v1g1	ATPase, H+ transporting, lysosomal V1 subunit G1
CTGCGGGTCT	7	0	0.009358827	Angptl7	Angiopoietin-like 7
GACCGTCTCA	0	9	0.0098287	Slc4a4	Solute carrier family 4 (anion exchanger), member 4
TTGGACTGAG	0	9	0.0098287	Gabarapl2	Gamma-aminobutyric acid (GABA-A) receptorassociated protein-like 2
TGATTTTGAA	9	1	0.01011074	Por	P450 (cytochrome) oxidoreductase
GAGACTAGCA	3	15	0.010247643	Tspan3	Tetraspanin 3
GTCGTGCCAT	14	33	0.010481768	Nudt19	Nudix (nucleoside diphosphate linked moiety X)-typemotif 19
TGAGGGGAGC	1	12	0.011021395	Flrt2	Fibronectin leucine rich transmembrane protein 2
TGCCCCCTCC	1	12	0.011021395	Cgnl1	Cingulin-like 1
TAGCTTTAAA	74	45	0.011671747	Igfbp7	Insulin-like growth factor binding protein 7

Mitochondrial, unknown, and ambiguous tags (those that match multiple genes) were excluded from the analysis.

**Table 3:** Genes which are differentially expressed between ROP-Os/+ and C57-Os/+ which have been shown to be expressed in the glomerulus

**Gene symbol**	**ROP-Os**	**C57-Os**	**P-Value**
Glud1	54	14	2.7461E-06
Pea15	15	0	0.000115878
Aspm	15	1	0.000577048
Hsp90ab1	0	14	0.001280824
Rpl23	14	39	0.0017137
Atpif1	19	4	0.002494576
Rpl37	19	4	0.002494576
Aqp1	1	13	0.007371123
Mdh1	36	66	0.008331722
Ndrg1	46	23	0.008962126
Atp6v1g1	7	0	0.009358827
Ctbp1	7	0	0.009358827
Nudt19	14	33	0.010481768
Cgnl1	1	12	0.011021395
Igfbp7	74	45	0.011671747
Cox6c	39	19	0.012221381
Cox4i1	26	10	0.012316032
Ddx5	27	11	0.013319653
Psap	53	30	0.018772506
Agps	5	0	0.020110086
Dpep1	5	0	0.020110086
Prdx6	5	0	0.020110086
Tmsb4x	5	0	0.020110086
Tufm	5	0	0.020110086
Uqcrh	5	0	0.020110086
Pdzk1ip1	7	20	0.022573009
Mdh2	12	3	0.022949534
Sod1	12	3	0.022949534
Id2	3	12	0.032439845
Sdf4	0	6	0.035034341
Tns1	14	14	0.037827945
Herpud1	16	6	0.038419946
Ttr 16	16	6	0.038419946
Prdx1	5	16	0.041379494
Calm2	11	3	0.042872521
Rps28	11	3	0.042872521
Slc25a3	14	28	0.042939254
Ankhd1	7	1	0.043285935
Fech 7	7	1	0.043521115
Tmem111	7	1	0.043285935
Gnb2	7	18	0.043285935
Abhd3	4	0	0.044128269
Acad9	4	0	0.044128269
Aig1	4	0	0.044128269
Atp6ap1	4	0	0.044128269
B3gat3	4	0	0.044128269
Cct4	4	0	0.044128269
Cited2	4	0	0.044128269
Creld1	4	0	0.044128269
Ctgf	4	0	0.044128269
Fads2	4	0	0.044128269
Fkbp2	4	0	0.044128269
HNMT	4	0	0.044128269
Itgb1	4	0	0.044128269
Kctd2	4	0	0.044128269
Mcl1	4	0	0.044128269
Mknk2	4	0	0.044128269
Mpdu1	4	0	0.044128269
Nucb1	4	0	0.044128269
Pmm1	4	0	0.044128269
Ptger4	4	0	0.044128269
Rab24	4	0	0.044128269
Sbf1	4	0	0.044128269
Scpep1	4	0	0.044128269
Sfrs6	4	0	0.044128269
Zdhhc8	4	0	0.044128269
Hspa5	3	11	0.047594628

### Assembly of differentially expressed networks and pathways

**Figure 1:**
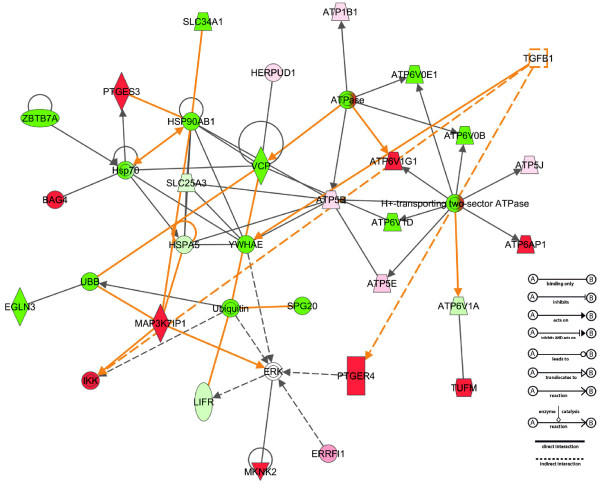
**Ingenuity Pathway analysis generated Network 1 diagram.** The networks are based on known protein-protein interactions and functional relations. Genes that are differentially expressed in the SAGE libraries are depicted in shades of red (upregulated) or green (downregulated); empty symbols represent network genes that were not differentially expressed in the SAGE libraries. TGFβ is inserted into the model to identify gene whose expression is modulated by TGFβ. The diagram to the right explains the relationship lines.

Using the Ingenuity Pathway Analysis software to perform a connectivity analysis (see Methods), we identified multiple molecular networks of genes differentially expressed in ROP-*Os*/+ and C57-*Os*/+ kidneys. These interactions are developed from published literature describing either physical or functional interactions between the molecules. The 13 most significant gene networks, based on connectivity and their imputed molecular and cellular functions, are shown in Table 4; corresponding diagrams of the actual connectivity networks are shown in Figure 1 and the supplemental data (Additional file 1).

**Table 4:** The rank list of the metabolic networks which encompass differentially expressed genes between ROP-Os/+ and C57-Os/+

**ID**	**Score**	**Focus Molecules**	**Top Functions**
1	61	32	Energy Production, Small Molecule Biochemistry, Genetic Disorder
2	55	30	Molecular Transport, Small Molecule Biochemistry, Cellular Function and Maintenance
3	44	26	Cellular Compromise, Cellular Assembly and Organization, Cellular Function and Maintenance
4	39	24	Cell-To-Cell Signaling and Interaction, Nervous System Development and Function, Cellular Assembly and Organization
5	33	21	Genetic Disorder, Neurological Disease, Metabolic Disease
6	23	17	Cellular Assembly and Organization, RNA Post-Transcriptional Modification, Protein Synthesis
7	19	14	Cellular Development, DNA Replication, Recombination, and Repair, Nucleic Acid Metabolism
8	19	14	Gene Expression, Genetic Disorder, Metabolic Disease
9	7	13	Carbohydrate Metabolism, Molecular Transport, Small Molecule Biochemistry
10	16	13	RNA Post-Transcriptional Modification, Cell Death, Post-Translational Modification
11	15	12	RNA Post-Transcriptional Modification, Cellular Function and Maintenance, Carbohydrate Metabolism
12	15	12	Carbohydrate Metabolism, Nucleic Acid Metabolism, Small Molecule Biochemistry
13	15	12	Genetic Disorder, Hepatic System Disease, Liver Cholestasis
14	14	11	Gene Expression, Lipid Metabolism, Molecular Transport
15	2	10	Cell Signaling, Infection Mechanism, Cell Death
16	2	1	Genetic Disorder
17	2	1	Genetic Disorder, Metabolic Disease, Lipid Metabolism
18	2	1	Molecular Transport, Protein Trafficking
19	2	1	Lipid Metabolism, Nucleic Acid Metabolism, Small Molecule Biochemistry
20	2	1	Cell Morphology, Cancer, Reproductive System Disease
21	2	1	Cancer, Cellular Development, Skeletal and Muscular System Development and Function
22	2	1	Genetic Disorder, Neurological Disease, Small Molecule Biochemistry
23	2	1	Lipid Metabolism, Small Molecule Biochemistry
24	1	1	Cancer, Genetic Disorder, Hepatic System Disease
25	1	1	DNA Replication, Recombination, and Repair, Cellular Compromise, Cell Death

Genes from the SAGE kidney libraries, which met the 2-fold difference in expression level cut-off between ROP-Os/+ and C57-Os/+ were assigned biological functions using the Ingenuity Pathways Knowledge Base. A right-tailed Fisher’s exact test was used to calculate a p-value describing the probability that each biological function assigned to that network is due to chance alone. Networks were ranked according to the combinatorial p-value of differentially expressed genes represented in the network. Corresponding network diagrams are shown as supplemental figures S3-S14.

**Figure 2:**
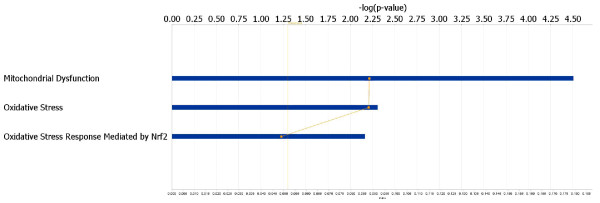
**Ingenuity Pathway Analysis identifies canonical pathways containing SAGE differentially expressed genes.** These pathways are ranked by their statistical significance (−log p-value) which is shown along the horizontal axis. The top three pathways are depicted in this figure, and highlight the prominence of mitochondrial dysfunction and oxidative stress genes which could potentially mediate renal injury in the ROP-*Os/+* mouse model.

Because TGFβ has been implicated in the pathogenesis of glomerulosclerosis, we next chose to overlay the canonical TGFβ signaling network onto our connectivity networks. TGFβ1 signaling pathway target genes, *Hnf4a*, *Itch, and Map3K7ip1,* were upregulated in the ROP-*Os/+* kidneys, in agreement with published microarray analysis of laser-captured/microdissected human glomeruli from FSGS biopsies [45]. Overlaying TGFβ1-regulated signaling pathways on to the connectivity networks of differentially expressed genes demonstrated that directional changes in gene expression patterns (up or down) in multiple networks (1, 2, 6, 8, and 13) could be a result of TGFβ1 signaling. Figure 2 shows molecular network 1 (Table 4) with the TGFβ signature identified.

We also performed a functional analysis using the Ingenuity Pathway Analysis system (see Methods). These data demonstrated functional clustering of differentially expressed genes within known canonical pathways, and highlighted mitochondrial dysfunction and oxidative stress responses as mechanisms for disease (Figure 2). Interestingly, a Nrf2-dependent stress response, which is the target for a new class of diabetic nephropathy drug [46], appears to also be activated in ROP-*Os/+* kidneys.

**Figure 3:**
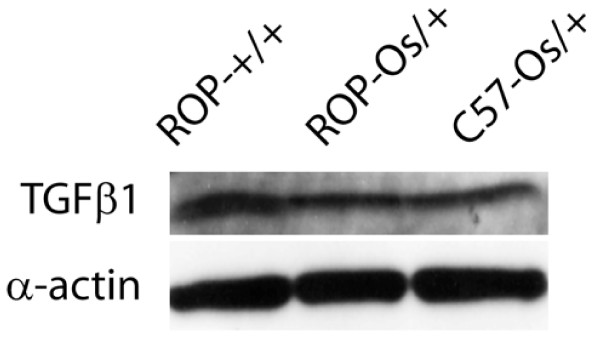
**A representative western blot analysis of protein extracts from 6 weeks old ROP-Os/+ and C57-Os/+ kidneys probed with anti-TGFβ antibody.** For comparison proteins from the WT ROP-+/+ kidneys are included. α-actin is used for loading control. Densitometry analysis of protein blots showed no difference in TGFβ protein level between ROP-Os/+ and C57-Os/+.

Surprisingly, TGFβ Tag was not differentially expressed between the sclerotic and non-sclerotic kidneys*.* Because the pathway analyses identified a prominent role for TGFβ in ROP-*Os/+−*dependent FSGS pathogenesis, we there fore compared TGFβ proteinbetween ROP-*Os/+* and C57-*Os/+* kidneys using Western blot analysis, and found no significant difference between the two strains (Figure 3).

### Itch overexpression increases sensitivity to TGFβ invitro

**Figure 4:**
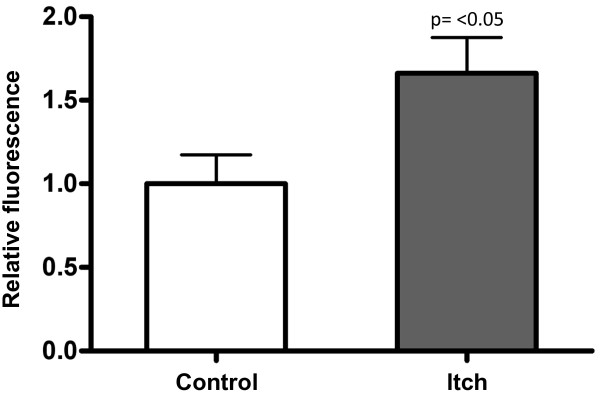
***Itch *regulation TGFβ signaling, as determined by PAI-1/luciferase activity in mink lung epithelial cells (seeMethods).** Cells were either transiently transfected with pCDNA3.1 empty vector (control) or mouse *Itch* cDNA in pCDNA3.1 under the control of a CMV promoter. Both groups were treated with TGFβ (200 ng/ml, 8 hr, 37°C). The readout (relative fluorescence), which is depicted on the Y-axis is calculated from the ratio of fluorescence units in cells transfected with *Itch* to fluorescence units in control cells (n = 3). Results are expressed as mean ± SD. A two-tailed *t*-test yielded a p-value of < 0.05 between groups.

To test the involvement of TGFβ-related candidate genes in glomerular fibrogenesis, we evaluated the effect of *Itch*, which was upregulated in ROP-*Os/*+ kidneys, on *TGFβ* signaling. This gene was selected because *Itch*, an E3 ubiquitin ligase, amplifies TGFβ signaling by facilitating the interaction between the TGFβ receptor and SMAD2 [47]. For these experiments we used mink lung epithelial cells which express the firefly luciferase under the control of minimal TGFβ-responsive PAI-1 promoter [38]. As seen in Figure 4 cells transfected with *Itch* cDNA showed higher luciferase activity in response to TGFβ, compared to control cells transfected with empty vector, suggesting that enhanced *Itch* expression may be relevant to the ROP-*Os/+* phenotype by stimulating TGFβ signaling.

### Differentially expressed genes in murine FSGS are similarly regulated in human FSGS

To determine if the comparative transcriptome analysis illuminated changes in gene expression in human FSGS, we compared the 40 most significant, differentially expressed genes derived from the ROP-*Os/+* vs. C57-*Os/+* analysis, with microarray data from kidney biopsy samples derived from patients with FSGS [48]. Of the 40 queried genes, corresponding oligonucleotides from six (*ACSM2A*, *TSPAN33*, *ERRFI1*, *NDUFA12*, *TMEM27*, and *RHPN2*) were not spotted on the array chip. Of the remaining 34 genes, 17 were concordantly regulated in the human glomerulus and ROP-*Os/+* mouse kidney, and altered mRNA expression in the human samples was statistically significant for 13 of the 17 genes. Two of the 13 genes were also concordantly regulated in the human tubulointerstitium and ROP-*Os/+* mouse kidney (Table 5). An additional concordantly regulated gene, *ID2*, which encodes inhibitor of DNA binding 2, localized exclusively to the human tubulointerstitium.

**Table 5:** Concordantly regulated SAGE transcripts from the ROP-Os/+ mouse model (as compared to C57-Os) and transcripts derived from microarray of human kidney biopsies from patients with FSGS (as compared to normal controls)

**TAG count in ROP**-**Os**	**TAG count in C57**-**Os**	**Genes concordantly regulated between mouse model and human Glomerulus**	**log Fold Change**	**q-value**	**Genes concordantly regulated between mouse model and human tubuloninterstitium**	**log Fold Change**	**q- value**
0	7	Slc 22a6	−0.42	0.00	Slc22a6	−0.22	0.00
0	9	Slc4a4	−0.32	0.00			
1.35	13	Aqp1	−0.30	0.00			
5.4	0	Psmb5	0.21	0.00			
5.4	0	Pcbp1	0.24	0.00			
5.4	0	Psma3	0.31	0.00			
6.75	20	Pdzk1ip1	−0.15	0.02			
74.25	45	Igfbp7	0.14	0.02			
12.15	3	Mdh2	0.16	0.02			
9.45	0	Vamp8	0.11	0.03			
8.1	1	Tmbim4	0.12	0.03			
5.4	0	Ndufb11	0.21	0.03			
4.05	16	Lifr	−0.09	0.05	Lifr	−0.27	0.00
12.15	3	Sod1	0.06	0.09			
16.2	6	Herpud1	−0.07	0.14			
2.7	13	Atp6v1a	−0.03	0.15			
8.1	42	Nrp2b	−0.03	0.15			
2.7	12		0.11	0.11	Id2	−0.40	0.00

## Discussion

Focal and segmental glomerulosclerosis is the histopathologic pattern of a spectrum of renal diseases which start with the glomerulus. There exists the likelihood that multiple insults converge on a common pathogenic pathway. Monogenic forms of familial FSGS due to mutations in TRPC6, α-actinin-4, podocin or APOL1 have been previously described [15,16,49]. To identify novel, candidate FSGS susceptibility genes, we compared SAGE-generated kidney transcriptomes from ROP-*Os/+* and C57-*Os/+*mouse strains that are glomerulosclerosis-susceptible and resistant, respectively. Both strains carry the *Os* mutation which causes reduction in nephron number. The quantitative decrease in nephron number is similar between the two strains, however, the C57-*Os/+* strain is protected against the development of renal disease [22,50]. The glomerulosclerosis phenotype that is associated with the *Os*-induced reduction in nephron number has been shown in other strains, but it appears the C57-BL/6 genetic background is unique in its resistance to glomerulosclerosis [23,24]. The increase in glomerular surface area in the ROP-Os/+ strain reflects a hyperfiltration physiology which appear to be not as prominent in the C57-Os/+ strain. Comparison between the ROP-*Os/+* and C57-*Os/+* transcriptome is therefore a useful approach to identify potential nephropathy genes.

### Potentialcandidate nephropathy genes and pathways

There are >100 potentially different expressed genes with a p-value of <0.05 between the mouse strains studied in this report. It is likely that the sclerosis phenotype of the ROP-*Os/+* mice (or the resistance of the C57-*Os/+* to sclerosis) can be attributed to the combined effect of multiple genes. The renal pathology is due to the expression pattern of these genes on top of the reduced nephron number with its consequent hyperfiltration. The FSGS phenotype is not merely due to difference in the expression of background genes, as we contrasted the wild type ROP versus C57BL/6 kidney transcriptome using microarray, and did not detect differential expression of the SAGE identified genes (data not shown).

In addition to the TGFβ-related genes, which were evaluated in detail in Results, SAGE data showed that neuropilin-2 (*Nrp*2), which is expressed in the glomerulus and regulates vascular endothelial growth factor (*Vegf*) signaling [51,52], is downregulated in the sclerosis prone ROP-*Os/+ *mouse. *Nrp*2 knockout mice show progressive glomerular damage after injection of the podocyte toxin, adriamycin, in contrast to their wild type littermates, who recover after the initial injury (J. Sedor, unpublished data).

*Pea*15 gene, which is overexpressed in the sclerotic mouse kidney, is a death effector domain-containing protein and promoter of autophagy. PEA15 inhibits caspase activation and increases ERK activity [53]. Transgenic mice overexpressing PEA15 have glomerular mesangial expansion and a histological pattern similar to diabetic nephropathy [54]. PEA15 induces the expression of the glucose transporter Glut1 in skeletal muscle cells [55]. Interestingly, Glut1 is also overexpressed in glomeruli from the FvB-*Os/+* mice (*Os* mutation bred on FvB genetic background) [24], suggesting that PEA15 regulation of Glut1 may play a role in non-diabetic glomerular scarring.

In the ROP-*Os/+ *strain a number of ubiquitin-proteasome pathway genes are differentially expressed; *Psmb*5, *Psma*3, *Fbxl12*, and *Itch* gene expression was enhanced, while *Siah1a *was downregulated. The differential expression of these genes suggests a role for aberrant protein degradation in glomerulosclerosis phenotype of the *Os* mice. *Itch* has been linked to the regulation of a multitude of signaling cascades, including TGFβ and EGF through ubiquitin and non-ubiquitin mediated mechanisms [47,56]. We tested the effect of *Itch* overexpression *in vitro* as a proof of concept that it regulates TGFβ signaling, which has been widely implicated in the pathogenesis of glomerulosclerosis. The experimental data support the hypothesis that *Itch* overexpression increases TGFβ signaling, independent of TGFβ ligand level (Figures 3 and 4). *Itch *expression is regulated by the Src kinase Fyn [57-60], which regulates podocyte function through phosphorylation of nephrin [61,62]. Bigenic *Fyn*/*Cd*2*ap *heterozygotes demonstrated an FSGS phenotype [63]. Because of the links with Fyn, TGFβ and other TGFβ-related signaling molecules, such as Pcbp1 [64], we speculate that *Itch* may function as a molecular rheostat, by regulating downstream TGFβ signaling (and FSGS pathophysiology) independent of ligand concentration.

Laser capture microdissection-microarray analysis of FSGS glomeruli demonstrated multiple changes consistent with activity of TGFβ signaling [45]. Although we did not identify differences in TGFβ ligand SAGE tag expression in our libraries, and Western blot analysis showed similar TGFβ protein expression between ROP-*Os/+ *and C57-*Os/+* mouse kidneys, we postulate that the TGFβ pathway in the ROP-*Os/*+ kidney is upregulated by downstream molecules, such as Itch and SnoN.

Using the Ingenuity Pathway Analysis engine, the clustering of genes involved in oxidative stress response like the *Nrf2* response genes and *GPX* suggests a role for an electrophile or oxidative stress in the mechanisms promoting renal injury in the ROP-*Os*/+ model [65]. Recently, bardoxolone methyl, which activates the Keap1-Nrf2 anti-oxidant pathway, was shown to protect kidney function in patients with type 2 diabetes [46].

Genes overexpressed in the C57-*Os/+* mouse may be protective. For example, glutathione-S-transferase theta (*Gstt*1) tag counts were 18.6 fold higher in the C57-*Os/+* compared to ROP-*Os/+* mice, and it was shown that the deletion of this gene increases the likelihood of ESRD in diabetic patients [66]. Also, upregulation of *Hsp90ab1*, *Vcp*, *Prdx1*, and *Prdx2* genes in the sclerosis resistant C57-*Os/+* strain could indicate a robust protective response to the increased oxidative stress induced by the *Os* mutation.

### Differentially regulated genes known to be involved in renal pathology

Connective tissue growth factor (*Ctgf*), integrin-β1 (*Itgb*1), and secreted phosphoprotein 1 (*Spp*1) were all upregulated in the sclerosis-prone ROP-*Os/+* mouse kidney. These genes are also upregulated in a other renal diseases characterized by fibrosis [67-72]. On the other hand, complement factor H (*Ctf*) and prosaposin (*Psap*) are relatively downregulated in ROP-*Os/+* kidneys, similar to observations in membranoproliferative glomerulonephritis (MPGN) and tubular damage [73,74].

### The ROP-Os/+ mouse and human FSGS

By comparing our ROP-*Os/+*SAGE libraries to previously published kidney SAGE libraries, we identified 13 genes, which were concordantly regulated in ROP-*Os/+* kidneys and human FSGS kidney biopsies, all of which localize to the glomerulus. None of these have been previously detected using either SAGE or microarray libraries from normal kidneys, ( http://www.ncbi.nlm.nih.gov/geo/) [43,75], suggesting that novel glomerular gene sets are induced as part of the pathophysiology of FSGS. Alternatively, the regulation of gene expression in fibrotic glomeruli could be a manifestation (rather than a cause) of fibrogenesis, though we attempted to minimize this possibility by selecting mice for study at an age prior to the appearance of glomerulosclerosis.

### Limitations of SAGE

SAGE is a powerful tool for gene and pathway discovery. However, like other global gene expression analysis methods, it has inherent limitations. The lack of a uniform annotation tool for SAGE tags makes data analysis burdensome. Although SAGE permits assembly of a comprehensive transcriptome, the sequencing costs can be significant. As a result, most labs have resorted to microarray technology to achieve adequate, but less comprehensive coverage.

## Conclusions

In this paper we have identified multiple candidate nephropathy genes which might regulate FSGS pathogenesis in a mouse model. The identified candidate genes and pathways need to be validated in knockout mice and/or other renal disease models, in order to discover targets for rational drug design or novel renal disease susceptibility markers. The number of networks derived from the differentially expressed gene set underscores the complexity of renal disease pathogenesis, and suggests that therapies directed at multiple pathways will be needed to effectively treat human kidney diseases.

## Competing interests

The authors declare that they have no competing interests.

## Authors’ contributions

AE-M carried out phenotyping of the mice, construction and analysis of SAGE libraries, confirmation of SAGE tags, IPA analysis, TGF-β assays, and manuscript preparation. JS participated in phenotyping, experimental design, and manuscript preparation. JS participated in phenotyping, experimental design, and manuscript preparation. SI assisted with database mining and experimental design. KG conducted statistical analysis of SAGE tags. VN and MK performed the comparative analysis of SAGE tags to human kidney disease genes. PH, SB, FP and EE participated in tag expression confirmation and SAGE tag annotation. All authors read and approved the final manuscript.

## Pre-publication history

The pre-publication history for this paper can be accessed here:

http://www.biomedcentral.com/1471-2369/13/61/prepub

## Supplementary Material

Additional file 1 Supplemental methods, real-time PCR primers sequences, and full table of statistically significant, differentially expressed SAGE Tags (Table 2S). Click here for file
