# Supplementary material for: Identification of nephropathy candidate genes by comparing sclerosis-prone and sclerosis-resistant mouse strain kidney transcriptomes
Source: BMC Nephrol. 2012 Jul 19;13:61. doi: 10.1186/1471-2369-13-61 (PMC3502501; doi:10.1186/1471-2369-13-61)
Supplement: Additional file 1 — Supplemental methods, real-time PCR primers sequences, and full table of statistically significant, differentially expressed SAGE Tags (Table 2S). [file 1471-2369-13-61-S1.pdf]

In order to verify that the sclerotic phenotype previously described in the ROP-Os mice is still expressed in our animal colony, we phenotyped the mice using biochemical and histological measures. Figure 1s shows a representative image of PAS stained kidney section from ROP-Os and C57-Os mice, there is increased PAS positive material in the ROP-Os compared to C57-Os.

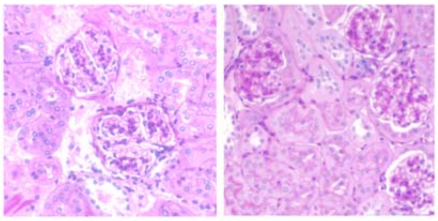

Figure 1s. Representative image showing PAS stained paraffin sections from C57-Os and ROP-Os mice showing increased PAS positive material in the mesangium of the ROP-Os glomeruli.

Urinary albumin measurement. Urinary albumin concentration was determined using a mouse albumin ELISA kit (Bethyl Laboratories, Montgomery, TX) according to the manufacturer's protocol. In brief, microtiter ELISA plates were coated with an affinity-purified goat anti-mouse albumin antibody, then washed and blocked. Subsequently, protein standards and unknown test samples were added to the plates and incubated for 1 hour. The plates were vigorously washed multiple times and then incubated with a different goat anti-mouse albumin antibody conjugated to HRP. After the incubation step, the colorimetric reaction was initiated using the TMB color substrate (Kirkegaard & Perry Labs, Gaithersburg, MD 20879). The color reaction was stopped by adding 2 M sulfuric acid and the color depth determination was measured at a wavelength of 450 nm. The standard curve was plotted using a semi-logarithmic scale and sample concentration was determined by comparison to the standard curve. Samples with concentration outside the range of the standard curve were diluted and re-assayed.

Urinary and serum creatinine measurements: Urine and serum creatinine were assayed by the enzymatic creatininase method using CREA-Plus reagent according to the manufacturer's protocol (Roche diagnostics; Indianapolis, IN). This assay has been shown to be more accurate in mice and correlates with results obtained by HPLC [1,2]. The ROP-Os/+ mice had higher albumin to creatinine ratio as early as 6 weeks.

Sclerosis score: Mesangial sclerosis was scored by three blinded observers and averaged using a previously described method. [3,4]. ROP-Os mice displayed a higher sclerosis score as previously described.

Confirmation of SAGE tag differential expression. To validate differential gene expression, we confirmed the relative mRNA concentrations of a selected number of genes using kidney RNA derived from different animals (average, n=3). We used multiple methods for confirmation based on abundance of the expression of the target cDNA, the presence of different isofroms or similar cDNAs, and the possibility of designing accurate real-time PCR primers.

<u>RNase protection assays</u> were carried out using the Direct Protect and Max-Script kit (Ambion, Austin, TX). Briefly, cDNA fragments were PCR-generated using intron-spanning, exon-specific primers. The PCR products were cloned into pCR-II-topo (Invitrogen, Carlsbad, CA). The recombinant plasmids were sequenced to ensure identity. [^{32}P] labeled cRNA probes were generated using the Max-Script kit and the probes were gel-purified according to manufacturer recommendation. The labeled probes were then annealed to the mRNA, followed by RNAse digestion. Protected probe fragments were resolved by electrophoresis and subjected to autoradiography using a phosphorimager, followed by quantification of the protected fragment intensity using ImageQuant software (Molecular Dynamics, Sunnyvale, CA). The signal from the protected probe is directly proportional to the abundance of the corresponding mRNA in the assayed pool. A *Gapdh* probe was used as a control to ensure equal input of mRNA across samples and normalize expression level.

<u>Real-time quantitative RT-PCR</u> was done using a Roche LightCycler PCR machine in combination with SYBR green reagent supplied by Roche (Roche, Indianapolis, IN) according to the manufacturer's protocol. The data obtained were normalized to either the *Gapdh* housekeeping gene or 18S rRNA. <u>Northern Blot analysis:</u> Northern blots were performed as previously described [5]. Briefly, [^{32}P] labeled cDNA probes generated by PCR were used to probe Northern blots using RNA obtained from both ROP-*Os/+* and C57-*Os/+* kidney cortices. Equal loading of RNA was ascertained by hybridization with a *Gapdh* cDNA probe. Signal intensity was quantified using densitometry in combination with ImageQuant software (Molecular Dynamics).

We selected the genes for confirmatory experiments based on the possible pathophysiologic and/or statistical significance. Among the genes we chose to confirm were *Gpx3* (glutathione peroxidase 3), *Nrp2* (neuropilin), and *Itch* (itchy E3 ubiquitin protein ligase homolog). All confirmatory approaches revealed relative expression levels in agreement with SAGE analysis.

Primers used in Real-time quantitative RT-PCR are depicted in table 1s.

Table 2s contains a listing of tags that are differentially expressed between ROP-Os and C57-Os mouse kidneys.

Gene	Upper Primer	Lower Primer
Nrp2	AGGACACGAAGTGAGAAGCC	GTGAGGGTTGAAGTTGAGAACA
Itch	CATGCCATCTACCGCCACTAC	TCGGCAGGTTCCAGTAACAAA
Siah1a	GGCCATCTTGTTTGTAGCAAC	TTTCGGTGTGTGGCAGAGTTATT
Pcbp1	AGACGCCTACTCGATTCAAGG	CGTGCATCATGGCAAAGTGA

Table 1s. Sequence of primers used in real-time PCR reactions

TAG	ROP-Os	C57-Os	P-Value	Gene Symbol
CTATCCTCTC	873	613	1.47E-10	Gpx3
ATTAACTTGG	54	14	2.75E-06	Glud1
TGGTTGCTGG	8	42	2.53E-05	Nrp2b
TCAAAAAAAA	15	0	0.000115878	Pea15
ACAAAAAAAA	20	2	0.000138392	Pde6c
AACTTGATTA	14	0	0.000237608	Ndufa12
GTTCACTTTC	27	6	0.000390333	Atp5e
CTGCTGTAAT	15	1	0.000577048	Aspm
TGTTGTGTTT	0	15	0.000858214	Lman2l
GTGAGCCCAT	0	14	0.001280824	Hsp90ab1
GCCACGCCCC	24	6	0.001296626	Hpd
ATTCTCCAGT	14	39	0.0017137	Rpl23
GTCCTGAGAG	9	0	0.002106134	Vamp8
ATAAAAAAAA	9	0	0.002106134	Bag4
ATTCTAACAT	15	2	0.002151531	Acadm
TGGATGCCTT	1	16	0.002228166	Adh1
GATTCCGTGA	19	4	0.002494576	Rpl37
GCTTTGAATG	19	4	0.002494576	Atpif1
TGCTGCTCCC	0	12	0.00287027	Gyk
GCTGGCCTCC	1	15	0.003314916	Rhoq
GCCAAGTGGA	22	6	0.0041826	Eef2
GTTTGTAAAA	22	6	0.0041826	Acsm3
AGATAACACA	8	0	0.004417209	Rere (atrophin-2)
AAGACCTATG	39	17	0.004902036	Dbi
ATCCGATTCC	11	31	0.005368014	Miox
GTCAATGACG	1	13	0.007371123	Aqp1
TCAGGCTGCC	180	130	0.008291421	Fth1
TTGTTAGTGC	36	66	0.008331722	Mdh1
CTAGTCTTTG	22	7	0.008750438	Rps29
CTGCTGTGGA	22	7	0.008750438	Hmgcs2
AGAGACAAGG	46	23	0.008962126	Ndrg1
GATCAGAAAA	7	0	0.009358827	Prdm16
GTGTGATACA	7	0	0.009358827	Pccb
CAGTTGGTTC	7	0	0.009358827	Mm.399814
CAGTAAAAAA	7	0	0.009358827	Map3k7ip1
AACTTTTAAA	7	0	0.009358827	Hp1bp3
GCTGTATTCA	7	0	0.009358827	Folh1
AATAAAAACT	7	0	0.009358827	FBXL12
TTTGTGACTG	7	0	0.009358827	Ctbp1
AGATCTGCCC	7	0	0.009358827	Atp6v1g1
CTGCGGGTCT	7	0	0.009358827	Angptl7
GACCGTCTCA	0	9	0.0098287	Slc4a4
TTGGACTGAG	0	9	0.0098287	Gabarapl2
TGATTTTGAA	9	1	0.010110742	Por
GAGACTAGCA	3	15	0.010247643	Tspan3
GTCGTGCCAT	14	33	0.010481768	Nudt19
TGAGGGGAGC	1	12	0.011021395	Flrt2
TGCCCCCTCC	1	12	0.011021395	Cgnl1
TAGCTTTAAA	74	45	0.011671747	Igfbp7
CAGCTTCGAA	14	3	0.012161366	Gstt2
TTGTGTGACC	14	3	0.012161366	8430408G22Rik
AATATGTGTG	39	19	0.012221381	Cox6c
CTAATAAAAG	26	10	0.012316032	Cox4i1

GCCTTCCAAT	27	11	0.013319653	Ddx5
TGGGCATCCA	5	19	0.014666055	Rpl26
CGTGCACGTG	0	8	0.014928188	Siah1a
GTGACTCACA	4	16	0.018307961	Lifr
AACTGAGGGG	53	30	0.018772506	Psap
TTCTAGCATA	49	27	0.019019108	Atp1b1
GGACTAGAGG	5	0	0.020110086	Uqcrh
CGATGCACTG	5	0	0.020110086	Uhmk1
GATAAAAAAA	5	0	0.020110086	Tufm
TCTGACAAAC	5	0	0.020110086	Tmsb4x
ACAGTCTCGC	5	0	0.020110086	Spcs2
TTCAAAAAAA	5	0	0.020110086	Smarcb1
AATCCATTTG	5	0	0.020110086	Slc13a1
CTACGGTTGC	5	0	0.020110086	Sidt2
TTCATTGTTT	5	0	0.020110086	Rhpn2
ACAGTGGGGA	5	0	0.020110086	Ptges3
GCTATAATAA	5	0	0.020110086	Psmb5
GACTATATAT	5	0	0.020110086	Psma3
TATTTTGTTT	5	0	0.020110086	Prnp
TACCTCCTAG	5	0	0.020110086	Prdx6
GATTTTATTT	5	0	0.020110086	Pcbp1
GAATCCAACT	5	0	0.020110086	Ndufb11
GTCCTATAAG	5	0	0.020110086	Mlf2
ACTCACCCTG	5	0	0.020110086	Ldhd
TATCTTGGCA	5	0	0.020110086	Hnrpl
GACAAGTTCC	5	0	0.020110086	Hint3 or 1700015F17Rik
TCTGAAAACT	5	0	0.020110086	Gsto1
GTCCTCAAAT	5	0	0.020110086	Glud1
GTTGAGGATG	5	0	0.020110086	Dpep1
CTCAGTAATG	5	0	0.020110086	Csrp2
TCGAACTAAG	5	0	0.020110086	Clec2h
AGCCTATGAT	5	0	0.020110086	Agps
CTGCAGAAAA	8	1	0.020870958	Upp2
CTATTAAAAT	8	1	0.020870958	Tmem37
GATATCATCA	8	1	0.020870958	Tmbim4
GAAACTTCTG	8	1	0.020870958	LOC545747
TTACATTGAA	8	1	0.020870958	Itch
CTTTAGAAAA	8	1	0.020870958	Errfi1
GCCACTGCCT	3	13	0.022098939	Tmem176a
TACTCTGTTG	3	13	0.022098939	Atp6v1a
TGCTGTGACC	7	20	0.022573009	Pdzk1ip1
ACTGCCGAGT	0	7	0.022793216	Slc22a6
AACTACAGCT	0	7	0.022793216	Serinc3
TATGTCAAGC	0	7	0.022793216	Rps12
AATAAACTCA	0	7	0.022793216	Mrpl32
TAACAACCAA	0	7	0.022793216	Cfh
AGAAACAAGA	12	3	0.022949534	Sod1
TCTTTGGAAC	12	3	0.022949534	Mdh2
TGAGCGCTGC	8	22	0.023778988	Pdzk1
ATCTGCTCAG	1	10	0.02480443	Nmb
GCATAGAAAT	12	28	0.02492426	Tmem27
TTGTGAGCCA	30	52	0.025647948	Acsm2
GCCCAGACCT	23	43	0.025882638	Sord
CAACTGTATT	20	8	0.027820704	Aco2

TGTGAACGAA	22	9	0.02997984	Cpt1a
AAAAAGTACC	23	10	0.031830932	ambiguous
GGGAGCGAAA	3	12	0.032439845	Id2
GGAAAAAAAA	9	2	0.032510072	Tspan33
GCACCGAACA	9	2	0.032510072	Dstn
TACACACACA	0	6	0.035034341	Tubb4
GCCCAGGTTT	0	6	0.035034341	Sdf4
CTAGATTGCG	0	6	0.035034341	Entpd5
AGAGTCAGCG	0	6	0.035034341	Atp6v0e
AAGTAAAGCG	1	9	0.037359878	Sec61g
AGACATACTG	4	14	0.037827945	Tns1
AATTCGCGGA	16	6	0.038419946	Ttr
TTAGCAGGAC	16	6	0.038419946	Herpud1
ATGTGGTGTG	5	16	0.041379494	Prdx1
CCGACGGGCG	5	16	0.041379494	ambiguous
GATGACACCA	11	3	0.042872521	Rps28
GCACAACTTG	11	3	0.042872521	Calm2
GACTGAATCT	14	28	0.042939254	Slc25a3
GTGATGTTTC	14	28	0.042939254	Cyb5
TTAAGCACTG	7	1	0.043285935	Tmem111
TCATTATTGA	7	1	0.043285935	Tgoln1
AACCCCTGCC	7	1	0.043285935	Ptms
TGACTATAGG	7	1	0.043285935	Osta
CTCACAAATG	7	1	0.043285935	Gng12
TTTCAAGAGT	7	1	0.043285935	Fech
TAATCTAGTG	7	1	0.043285935	Ankhd1
GGCAATGTGG	7	1	0.043285935	2310003F16Rik
GTTCTGACAA	7	1	0.043285935	1700020O03Rik
CCCACACTAC	7	18	0.043521115	Gnb2
AGTGCTTTGC	7	18	0.043521115	2310043N10Rik
ACAATAAACA	4	0	0.044128269	Zfp623
GCAGCGTCCG	4	0	0.044128269	Zdhhc8
TTGTACTTAA	4	0	0.044128269	Wdr75
TGGAAAGTGC	4	0	0.044128269	UBE3A,FBP2
CACAAAAGTC	4	0	0.044128269	Trim2
CATAATTGTG	4	0	0.044128269	Tmem53
AAACATTGGG	4	0	0.044128269	Tm9sf3
TATTTTAATA	4	0	0.044128269	Tloc1
ACCCAATTAT	4	0	0.044128269	St13,Tpm4
AATTAGAGAG	4	0	0.044128269	Spp1
TAATAAAATT	4	0	0.044128269	Slc7a8,Atf2
AAATAAACTT	4	0	0.044128269	Sirt3
AGCTAGGGCC	4	0	0.044128269	Sipa1l1
AGTGTGAAAC	4	0	0.044128269	Sfrs6
ACCCGCACTT	4	0	0.044128269	Scpep1
GTACAGCCCT	4	0	0.044128269	Sbf1
GCCGAGGAAG	4	0	0.044128269	Rps12
TAGCCTTGTC	4	0	0.044128269	Rg9mtd1
AGGAGGGGTA	4	0	0.044128269	Rab24
ATGGCTTAAT	4	0	0.044128269	Ptp4a1
CAAAGAAAAA	4	0	0.044128269	Ptger4

GAAAACATTA	4	0	0.044128269	Psat1
TCAGCAGAGG	4	0	0.044128269	Prkab1
AGGATGCTTG	4	0	0.044128269	Ppp2ca
GGAGTTGTGC	4	0	0.044128269	Pmm1
CAGTACAAAT	4	0	0.044128269	Pigx
TGTATGTCTT	4	0	0.044128269	Pfkp
AAGAAAGGAG	4	0	0.044128269	Pcolce
AAAAAATGGA	4	0	0.044128269	Pccb
GCCCCAGGAG	4	0	0.044128269	Nucb1
GAGTTTTCAC	4	0	0.044128269	Nadk
TCCTCCAGTC	4	0	0.044128269	Mucdhl
TCTTACTCTT	4	0	0.044128269	Mpv17l
GTTTTAATTG	4	0	0.044128269	Mpdu1
CAAGCACTTT	4	0	0.044128269	Mknk2
GGAAAAGTGA	4	0	0.044128269	Mcl1
CGCTGATAGG	4	0	0.044128269	Kctd2
TTATTAAAAT	4	0	0.044128269	Jam2,Pfdn4
TGCAGGTGCA	4	0	0.044128269	Itgb1
AATAAACGCC	4	0	0.044128269	Imp4
AACATTCTCT	4	0	0.044128269	Ikbkg
ATCACAGGTG	4	0	0.044128269	Igfbp4
AGCTGGTGCT	4	0	0.044128269	Igfals
TATATGCACT	4	0	0.044128269	HNMT
ACCTTCACAC	4	0	0.044128269	Hnf4a
CTGAATATGG	4	0	0.044128269	H2-Ke6
AAGCTGCTTG	4	0	0.044128269	Grpel1
GGAAGATGAA	4	0	0.044128269	Grin2a
CTTTTGTTTT	4	0	0.044128269	Gats
CACTACACGG	4	0	0.044128269	Fkbp2
GTGCAAACTC	4	0	0.044128269	Fgg
AGCCAAGAGA	4	0	0.044128269	Fads2
TCTTAAATAT	4	0	0.044128269	Dld
TTTGCACCTT	4	0	0.044128269	Ctgf
CTGCCAGCTC	4	0	0.044128269	Creld1
TGTTCTGTCA	4	0	0.044128269	Clcnkb
AAAAGATACT	4	0	0.044128269	Cited2
GGAAAAAAAT	4	0	0.044128269	Chpt1
CCCAAGGAGA	4	0	0.044128269	Cct4
CGATCCCCTT	4	0	0.044128269	B3gat3
AACTTGAGGT	4	0	0.044128269	Atp6ap1
GCTGCTGTTA	4	0	0.044128269	Aspa
GCAGAGACTA	4	0	0.044128269	Asl
ATACTGACTT	4	0	0.044128269	ambiguous
TTCCAGTCTT	4	0	0.044128269	ambiguous
GTTTTGGATT	4	0	0.044128269	ambiguous
CAATCGTGAC	4	0	0.044128269	Aig1

TTTTTTTAAG	4	0	0.044128269	Acadl,Ncbp2
ACCGTGAGGG	4	0	0.044128269	Acad9
CTTATTTTTA	4	0	0.044128269	Abhd3
ATTTGATATT	4	0	0.044128269	2810407C02Rik
ATAACAGATT	4	0	0.044128269	2610204K14Rik
AAGCAGCAGC	4	0	0.044128269	Omg
AATTAGTTGT	19	8	0.045348967	Atp5j ATP
AGAAGACAGA	19	8	0.045348967	Abcb6 ATP
CAGGCCACAC	53	33	0.045918906	Atp5b
GTTCAGTCAA	3	11	0.047594628	Sult1d1
ATGCTTTTCA	3	11	0.047594628	Kap
TGGTGTAGGA	3	11	0.047594628	Hspa5
TGGTGTAAGC	3	11	0.047594628	Adh1
TGCAGTATTT	19	35	0.047766609	G6pc,
CCAAAAAAAA	20	9	0.04794967	Psmb3

Table 2s. Table showing statistically significant and annotated SAGE tags . Ambiguous tags are these corresponding to more than one gene and tags not corresponding to a known gene are excluded

Reference List

1. Palm M, Lundblad A: **Creatinine concentration in plasma from dog, rat, and mouse: a comparison of 3 different methods.** *Vet Clin Pathol* 2005, **34:** 232-236.

2. Keppler A, Gretz N, Schmidt R, Kloetzer HM, Groene HJ, Lelongt B *et al.*: **Plasma creatinine determination in mice and rats: An enzymatic method compares favorably with a high-performance liquid chromatography assay.** *Kidney Int* 2007, **71:** 74-78.

3. Bidani AK, Griffin KA, Plott W, Schwartz MM: **Genetic predisposition to hypertension and microvascular injury in the remnant kidney model.** *J Lab Clin Med* 1993, **122:** 284-291.

4. Zheng F, Striker GE, Esposito C, Lupia E, Striker LJ: **Strain differences rather than hyperglycemia determine the severity of glomerulosclerosis in mice.** *Kidney Int* 1998, **54:** 1999-2007.

5. Sambrook J, Fritsch EF, Maniatis T: *Molecular cloning: a laboratory manual*, 2nd edn. Cold Springs Harbor: Cold Springs Harbor; 1989.
